# Improving nutritional status of children using artificial intelligence-based mobile application postsurgery: randomised controlled trial

**DOI:** 10.1136/bmjnph-2023-000645

**Published:** 2023-12-02

**Authors:** Maryam Zahid, Ume Sughra, Sehrish Mumtaz, Mawara Hassan

**Affiliations:** 1 Armed Forces Institute of Cardiology and National Institute of Heart Diseases, Rawalpindi, Pakistan; 2 Al Shifa Research Centre, Al-Shifa Trust Eye Hospital Rawalpindi, Rawalpindi, Pakistan

**Keywords:** Nutrition assessment, Malnutrition, Nutritional treatment, Weight management, Nutrient deficiencies

## Abstract

**Background:**

Malnutrition is a common problem in children postsurgery especially in low-middle-income countries. Health-based mobile apps play an important role for better nutritional status.

**Methods:**

This is a two-arm randomised controlled trial, which was conducted at a tertiary care hospital, Rawalpindi. The study duration was 6 months from February 2021 to July 2021. The sample size with power of 80% and significance level of 5% was calculated to be 88. The intervention group was given a diet-related mobile app, and the usual care group was handed a pamphlet with diet instructions on discharge.

**Findings:**

The mean weight of all participants was 15 (SD 5.7) kg at the time of discharge. However, at the end of the 8th week, the mean weight of the participants in the usual care group was 16.5 (SD 7.2) kg and that of the intervention group was 17.1 (SD 5) kg. The average calorie consumed by the usual care group was 972 (SD 252) kcal and 1000.75 (SD 210) kcal by the intervention group after 8 weeks of discharge. The average protein consumed by the usual care group was 34.3 (SD 12.5) g and 39 (SD 6.4) g by the intervention group after 8 weeks of discharge.

**Interpretation:**

This study showed strength for the future of scalable modern technology for self-nutrition monitoring. There was a slight increase in the weight and nutritional intake of both groups.

**Trial registration number:**

NCT04782635.

**WHAT IS ALREADY KNOWN ON THIS TOPIC**

- Malnutrition and lack of nutrition education is a significant problem in low-middle-income countries especially in Pakistan.
- There is a paucity of relevant available literature on assessing the effects of mhealth, particularly in the context with postoperative children’s nutritional status particularly in Pakistan.

**WHAT THIS STUDY ADDS**

- This study focuses on the effects of nutrition-based mobile application on nutritional intake of users with low literacy, low financial status and postsurgical nutritional requirements.
- As a mobile health application provides a large amount of health information, users are often exposed to ambiguous or controversial information. This study adds a considerable investigation of the effects of these nutritional-based mobile application was required, as poor-quality, unproven apps contributing to a fragmented mHealth landscape will affect the nutritional status of the population.

**HOW THIS STUDY MIGHT AFFECT RESEARCH, PRACTICE OR POLICY**

- Pakistan is a developing country where people neither have sufficient healthcare facilities nor a strong infrastructure that can address the nutritional health needs of the people. Nutrition-based mobile application—the provision of diet planning and tracking nutritional intake through mobile will revolutionise the way healthcare is delivered. It will impact every aspect of health systems.
- It will offer exciting new prospects for future mHealth projects not only in the field of nutrition but also in other health-related fields, and developers must be encouraged to develop more innovative products.

## Introduction

Children after surgery have reduced energy intake and increased metabolic demands so they are at a risk for both acute and chronic malnutrition.^1^ In developing countries, the incidence of malnourishment is already high as in Pakistan 17.7% of the children suffer from wasting.^2^ A study conducted in a hospital of Rawalpindi showed 21% of the children with congenital heart disease (CHD) had in-hospital mortality, 47% among them where underweight and 49% were severely underweight.^3^ Pakistan launched the ‘Lady Health Workers (LHWs) Programme’ in 1994, the deliverables by LHWs somehow was affected due lack of staff. Programmes launched in low-income and middle-income country (LMIC) such as Pakistan are mostly unable to achieve the target due to lack of resources.^4^ App‐based mobile health interventions are favourable method of changing nutrition behaviours and nutrition‐related health outcomes.^5^ The example of successful mHealth-based service in Pakistan is an app called ‘e-Vaccs’. It was introduced in the province of Punjab to track the movement of vaccinators using GPS.^6^

There is a paucity of relevant available literature on assessing the usefulness of mhealth, particularly in the context with postoperative infants and young children nutritional status particularly in Pakistan,^7^ which signifies its importance in implementing such interventions in poor resource settings.

The objective of this research was to assess the effect of artificial intelligence on the nutritional status of children after cardiac surgery in comparison to the usual care group. We also aimed to assess the usefulness of a diet-related mobile app in comparison to the usual care group.

## Methods

This was a single-centre trial conducted at Armed Forces Institute of Cardiology and National Institute of Heart Disease in Rawalpindi, Pakistan.

### Trial design

It was a parallel group trial design and was registered with the ClinicalTrials.gov under trial identity number NCT04782635. The total study duration was 6 months from February 2021 to July 2021. Patient recruitment took 10–14 weeks (at least 7–8 patients per week) while intervention was given for 8 weeks per patient.

### Eligibility criteria

**Table:** 

**Inclusion criteria**	**Exclusion criteria**
Children of age 2–12 years were included in the study. Surgeries within first and second class of RACHS-1 Score were included (Risk Adjustment for Congenital Heart Surgery score was created to determine children <18 years risk of hospital mortality after undergoing CHD surgery). Respondents with smart phones and internet facilities were included in the study. Patients with caregivers who can read English were included in the study Patients discharged from the hospital were selected	Patients with multiple congenital abnormalities were excluded from the study Patient who were rehospitalised were excluded from the research.

### Intervention procedure

- Caregivers (mothers/fathers/guardians) of the patient were selected through permuted block and explained the procedure of intervention and written informed consent was requested.
- Mobile application was installed on their mobile.
- Application then calculated the macronutrient and micronutrient requirements of the child and made a standard diet plan for the child according to the requirements. The child can also change the plan according to the likes and dislikes.
- Then the child with help of the caregiver follows the diet plan created by the mobile application. Participants also marks the meals consumed on the application. This calculates the nutrients consumed and helps the user to monitor the dietary goals.
- After 8th week patient and caregivers visited the doctor for routine check-up, during the visit their nutritional status was assessed.

### Usual care procedure

- Caregivers were selected through permuted block and handed over pamphlet with dietary instructions on discharge. Then written informed consent was requested
- After 8 weeks, the patient and the caregiver visited the doctor for routine check-up, during the visit, weight and usual dietary intake were noted.

### AI based mobile based application ‘Eatbaby’

EatBaby is a SaaS product. Mobile application is specifically developed to improve and maintain nutritional status of children. This m health is designed to cater the dietary needs of children with different health problems including diabetes, cancer, coeliac disease and postoperative patients. This study only focused on postoperative patients with cardiac disease This application is used by any person who wants to search recipes, diet plans, awareness about healthy diet in terms of blogs and recommended diet plans according to their health goals. They can also create a diet plan by themselves. This application also helps to manage multiple profiles, for example, a mother wants to monitor and manage the diet of her two children along with herself. So she can create three profiles in the application.

## Applications elements

The platform consists of four parts as follows:

1. API application
  This is the core application of EatBaby. It contains all business logic and database connectivity. Both Web UI and mobile applications will use this API application.
2. Mobile application
  This application is used by those users who want to manage and plan their diet and their childrens. This application consumes the API application for data processing and storage.
3. Web UI application
  This application contains website pages and a web copy of the mobile application with web UI. This application only contains HTML/JS/CSS and call api functions for database operations.
4. Web admin application
  This application is used by the system admin where they can create food items, recipes and diet plans.

### Outcomes

Primary outcome of the study was to assess the effect of artificial intelligence on the nutritional status of children after cardiac surgery and usefulness of mobile application among children post cardiac surgery in comparison to the usual care group.

Weight changes in kilograms (kg), average caloric intake in kcal and average protein intake in grams was observed in both groups for 8 weeks.

Average carbohydrates intake in grams, average fats in grams and average vitamins and minerals intake in mg and µg was observed in both groups for 8 weeks.

### Sample size

Sample size was calculated using results from a previous study, according to Saleem *et al*,^8^ there was 12% reduction in wasting in the intervention group by the end of the study. Keeping a power of 80% and a significance level of 5% and to allow for a predicted 25% drop-out, sample size was estimated to be 88 (44 in each group).

### Randomisation

The random assignment of recruited participants into the two arms was performed by the research supervisor using web-based permuted block randomisation (4×8), ensuring 1:1 ratio between the two arms. Allocation concealment was conducted by the sequentially numbered opaque envelopes.

### Blinding

In this parallel clinical trial design, patients using usual care were blinded from the patient using the mobile-based application. The research supervisor who generated the random allocation sequence was blinded from the patients enrolled in trial by the principal investigator.

### Statistical analysis

Data were analysed by using SPSS V.21. The total sample size was 80 which was randomised for the intervention phase. Frequencies and percentages were calculated for categorical variables such as patient’s gender, caregiver’s education and perceived smart phone knowledge of the caregiver. Means and SD were calculated for continuous variables, such as age, weight and income. Independent sample t-tests used to find association of monthly income of participant’s caregiver, age of patient and height with both groups(intervention and usual care group). χ^2^ test was applied to find out association of caregiver’s education leavel, weight (preintervention and postintervention, gender, perceived smart phone knowledge of caregiver with both groups.

## Result

**Figure 1:**
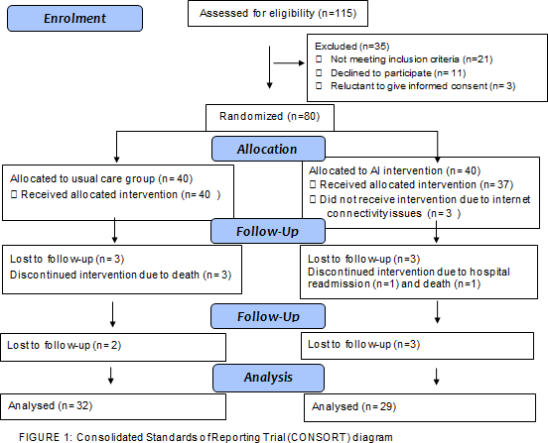
Consolidated Standards of Reporting Trial diagram. AI, artificial intelligence.

Total surgeries performed from February 2021 to May 2021 were 142, among those 120 surgeries were included in Risk Adjustment for Congenital Heart Surgery (RACHS) scores 1 and 2. A total of 115 participants were approached for the study and 61 participants completed the study. The demographic data of the intervention and usual care group is shown in table 1 whereas the Consolidated Standards of Reporting Trial diagram in shown in figure 1.

**Table 1:** Baseline characteristics

	Intervention group	Usual care group	P value
Age (mean)	59±36 months	56±30 months	0.747
Gender			0.298
Male	66% (N=19)	75% (N=24)	
Female	44% (N=10)	25% (N=8)	
Height	102±26 cm		0.429
Weight			0.003
≥−3 (Severely underweight)	3.0% (N=1)	41% (N=13)	
−2.99 to −2.0 (moderately underweight)	45% (N=13)	25% (N=8)	
−1.99 to −1.9 (normal weight)	52% (N=15)	34% (N=11)	
>2.0 (overweight)	0	0	
Education level of caregiver at the time of intervention			0.77
Secondary education	33.3% (N=1)	66.6% (N=2)	
Matriculation	42% (N=13)	58% (N=18)	
Intermediate education	50% (N=5)	50% (N=5)	
Bachelors	64% (N=7)	36% (N=4)	
Postgraduate education	50% (N=3)	50% (N=3)	
Smartphone knowledge			0.249
Little knowledge	31% (N=4)	69% (N=9)	
Moderate knowledge	50% (N=22)	50% (N=22)	
Good knowledge	75% (N=3)	25% (N=1)	
Mean monthly income	34870±15 269 rupees (US$219/month)		0.885

### Weight changes in participants of usual care and intervention group

Weight changes in both groups from discharge to the end of intervention, that is, 8th week is shown in table 2.

**Table 2:** Weight changes from discharge to 8th week in both groups

Weight	On discharge (preintervention)			After 8th week		
	Usual group	Intervention group	P value	Usual group	Intervention group	P value
Severely underweight	41% (N=13)	3.0% (N=1)	0.003	19.0% (N=6)	0% (N=0)	0.039
Moderately underweight	25% (N=8)	45% (N=13)		31% (N=10)	28% (N=8)	
Normal weight	34% (N=11)	52% (N=15)		50% (N=16)	72% (N=21)	
Overweight	0	0		0	0	
Total	32	29		32	29	

### Nutrients consumption in usual care and intervention group

Nutrients (calories, carbohydrates, proteins, fats, vitamins and minerals) consumption in intervention group was automatically generated by mobile application as the caregiver/patient marks the meal consumed on the application, whereas in usual care group patients/caregiver were interviewed for usual dietary intake. Then nutrients consumption was calculated using food composition table.

**Figure 2:**
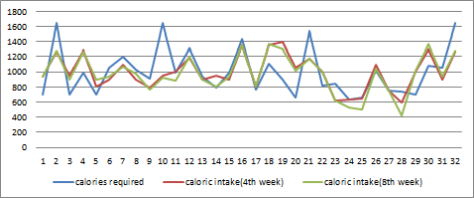
Calories consumption by participants in usual care group.

**Figure 3:**
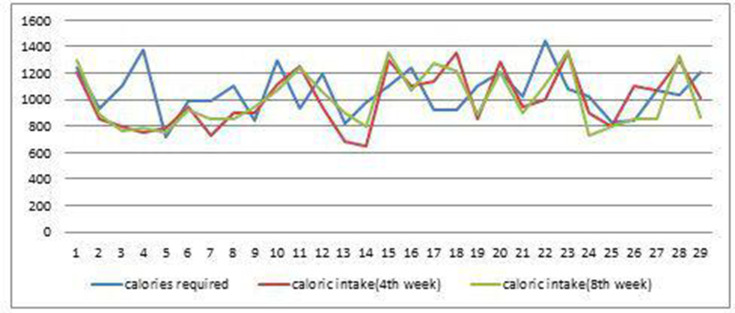
Calories consumption by participants in intervention group.

Mean calories required by all the participants were 1025±252 kcal. In our study, average calories consumed by the usual care group was 972±252 kcal after 8 weeks of discharge whereas average calories consumed by the intervention group was 997±206 kcal after 8 weeks of installation of mobile application. Details of the caloric intake in both groups are shown in figures 2 and 3.

Mean proteins required by all the participants were 44±10.9 g. In our study, average proteins consumed by the usual care group was 34.3±12.5 g throughout 8 weeks after discharge whereas average proteins consumed by the intervention group 39±6.4 g after 8 weeks of installation of mobile application.

Mean fats required by all the participants was 30±7.6 g. In our study, average fats consumed by the usual care group was 32.5±8.1 g throughout 8 weeks after discharge whereas and 31.4±5.4 g after 8 weeks of installation of mobile application.

Mean carbohydrates required by all the participants was 140±39 g, average carbohydrates consumed by the usual care group was 136±37 g after 8 weeks of discharge whereas average carbohydrates consumed by the intervention group was 136.6±62.9 g throughout 8 weeks of installation of mobile application .

Mean intake of vitamins and minerals among patients of usual care group and intervention group is discussed in table 3.

**Table 3:** Consumption of minerals and vitamins in intervention and usual care groups

Minerals	Required	Usual care group consumption	Intervention group consumption	Vitamins	Required	Usual care group consumption	Intervention group consumption
		8th week	8th week			8th week	8th week
Calcium	750±251 mg	758±402 mg	729±490 mg	Vitamin A	384±95.4 µg	209±192 µg	350±120 µg
Potassium	3609±538 mg	1466±642 mg	2760±600 mg	Vitamin C	17±12.8 mg	11.5±1.2 mg	13.5=±6.0 mg
Iron	8.5±1.4 mg	4.38±1.7 mg	7.3±2.5 mg	Vitamin D	5±0 µg	2.7±2.4 µg	4.6±4.1 µg
Sodium	1154±4.1 mg	1196±547 mg	970±263 mg	Vitamin E	7.1 ± 1.7 mg	3.4±3.1 mg	6.9±4.5 mg
Chromium	14.5±4.7 µg	35±5.8 µg	41±6.6 µg	Vitamin B_1_	0.6±0.12 mg	3.2±3.6 mg	1.5±2.1 mg
Copper	443±122 µg	160±98.6 µg	314.5±85 µg	Vitamin B_2_	0.6±0.12 mg	2.9±1.2 mg	2.1±0.6 mg
Iodine	95.6±11.9 µg	12.4±1.8 µg	56.3±12.6 µg	Vitamin B_3_	7.7±1.9 mg	2.8±3.5 mg	4.9±6.5 mg
Iodine	95.6±11.9 µg	12.4±1.8 µg	56.3±12.6 µg	Vitamin B_5_	2.8±0.7 mg	1.1±1.5 mg	1.2±2.5 mg
Manganese	1.45±0.24 mg	0.55±1.3 mg	1.05±1.02 mg	Vitamin B_6_	0.61±0.15 mg	1.9±5 mg	1.2±1.6 mg
Magnesium	128±54 mg	740±252 mg	410±210 mg	Vitamin B_7_	11.7±4.1 µg	7.9±2.6 µg	8.5±2.1 µg
Molybdenum	21.8±5.1 µg	0	9.1±6.5 µg	Vitamin B_9_	190±50 µg	118±29.8 µg	178±95 µg
Phosphorus	593±270 mg	974±252 mg	1031±390 mg	Vitamin B_12_	1.1±0.3 µg	3.9±4.5 µg	10.7±2.1 µg
Selenium	27.3±6.7 µg	39±13.7 µg	41±6.4 µg				
Zinc	4.7±1.7 mg	25±4.7 mg	15±2.1 mg				

## Discussion

In our study, intervention group caregivers were provided with a nutrition-based mobile application by a dietitian and usual care group was handed a pamphlet with dietary instructions on discharge by a dietitian. According to Källander *et al* review, in LMICs, reflecting impact from local conditions, reported infrastructural and technical limitations to implement mHealth ranging from low network capacity, inadequate distribution of signal strength, and low access to mobile phones to specific technical barriers, such as delay in database updating.^9^ Our study carried out in a tertiary care hospital in Rawalpindi with patients from all across Pakistan reported similar infrastructural and technical challenges including poor internet connection.

Malnutrition is high in children with CHD in various countries. However, heterogeneity exists from country to country implying differences in risk factors for malnutrition among children, in the different settings.^10^ In our study, the underweight patients were more than 80% of the population.

In our study, weight changes were studied in both groups, that is, intervention group and usual care group, although average weight of participants in both group was increased there was not much difference in average weight of both groups. Shah *et al* 11 reported a 6-month intervention involving healthy Swedish children aged 4.5 years via a smartphone application that provided educational information, addressed primary outcomes. The authors found no difference between the intervention and control group concerning anthropometrics. According to him, this mHealth interventions period was limited and these interventions did not fundamentally change the anthropometric measurements.

In terms of education level, users with higher education levels have a higher tendency to use mobile health applications.^12^ Similarly, in this study, most of the participants who used mobile application as the tool for dietary planning for their children were educated till 10th grade.

As for income, according to Post *et al*, the possession of smartphones and the adoption of mobile applications have a positive relation with income status. Thus, income is one of the important predictors for people who use mobile devices to promote health.^13^ All the participants in this study had low to moderate income status; however, those who used mobile application as the tool for dietary planning had average income status.

### Strength

With the rapid development of mobile internet technology and the popularity of intelligent terminal mobile devices, mobile health applications have exploded as the most direct tools for public personal health management. There are currently no dietary monitoring applications that meet the literacy and requirements of children postsurgery.

### Limitations

The use of applications for nutrition education is relatively new, and there is much research to be done. Mobile Health interventions period was limited, it is suggested that the duration of interventions measuring anthropometrics extend longer.

## Conclusion

This study showed strength for the future of scalable modern technology for self-nutrition monitoring. The average income status and education level of the respondents was low to average, it represents a population that has been slow to adopt new technology but our population showed interest in mobile application and used the technology after a brief training session. There was slight increase in the weight and nutritional intake of both groups as mobile. However, to provide lifestyle changes that may ultimately result in healthier body, it is suggested that the duration of interventions measuring anthropometrics extend longer.

10.1136/bmjnph-2023-000645.supp1 Supplementary data

## Data Availability

Data are available on reasonable request. Not applicable.
