# Supplementary material for: Improving nutritional status of children using artificial intelligence-based mobile application postsurgery: randomised controlled trial
Source: BMJ Nutr Prev Health. 2023 Dec 2;6(2):367–73. doi: 10.1136/bmjnph-2023-000645 (PMC11009550; doi:10.1136/bmjnph-2023-000645)
Supplement: Supplementary data [file bmjnph-2023-000645supp001.pdf]

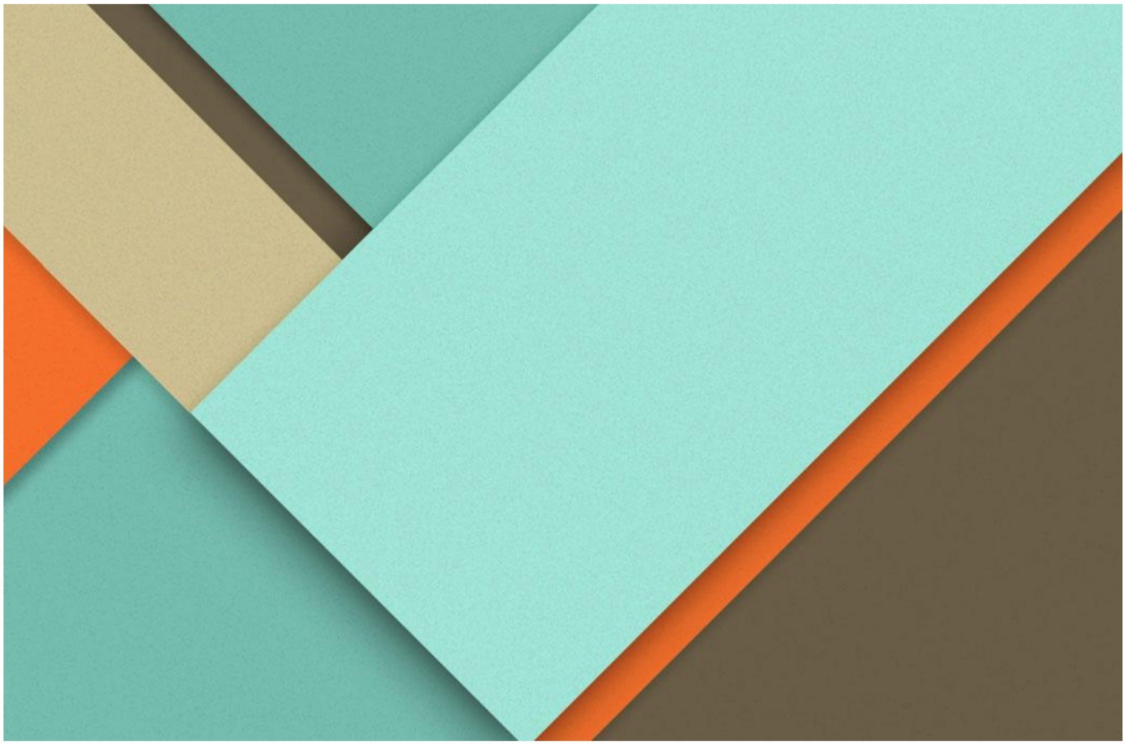

EatBaby

V 2.0.0

Technical Documentation

FBM SOLUTIONS (PVT) LTD.

Pakistan

www.fbm-solutions.com

EatBaby Technical Documentation

About this document: Aim of this document is to provide an overall understanding of the general functionality of the project. This document is an accompanying document for other documentation that we have and it should be used in conjunction with sitemap and other tabular documents. In order to be able to fully understand the site functionality the sitemap document should be studied in detail together with this document.

Confidentiality: All details on this document and any other information for this project are confidential.

BMJ Publishing Group Limited (BMJ) disclaims all liability and responsibility arising from any reliance placed on this supplemental material which has been supplied by the author(s)

Introduction

About the Product

EatBaby is a SaaS product. This application is used by any person who wants to search recipes, diet plans, awareness about healthy diet in terms of blogs and recommended diet plans according to their health goals. Users can also make their accounts and plan/manage their diet as well as the diet of their beloved-ones. They can also create a diet plan by themselves. This application also helps to manage multiple profiles, e.g. a mother wants to monitor and manage the diet of her two children along with herself. So she can create three profiles in the application.

Applications Elements

The platform will consist of 4 parts.

1. **API Application**

This is the core application of EatBaby. It contains all business logic and database connectivity. Both Web UI and mobile applications will use this API application.

2. **Mobile Application**

This application is used by those users who want to manage and plan their diet and their childrens. This application consumes the API application for data processing and storage.

3. **Web UI application**

This application contains website pages and a web copy of the mobile application with Web UI. This application only contains HTML/JS/CSS and call api functions for database operations.

4. **Web Admin application**

This application is used by the system Admin where they can create food items, recipes and diet plans.

Entities of the Platform

Within the EatBaby platform, there are several different entities and actors. This section is trying to give a brief description of each as an introduction and with the purposes of making the rest of the document easier to understand.

Food Items: These are the basic entity of the system, all calculations are based upon food items. Major attributes of food items are Name, Description, Image, nutrition facts, food category and serving information.

Recipe: Recipe is built by using different food items in different serving sizes. Major attributes of recipes are Recipe Name, Description, Image, Food items along with serving quantity and recipe category.

Food Time: Food time is the duration in which the user will consume a meal. Food Items are breakfast, lunch, dinner etc. We have a maximum of 6 food times. Each user can modify his time based on their needs and the user can delete a food time but cannot create a new one.

Diet Plan: This is the meal plan for a specific number of days. This diet plan does not contain start and end dates. Diet Plan can be created by using Food Items, Recipes along with the quantity of recipe.

Diet Plan User Child: This is the everyday diet plan of a user child. This diet plan is derived from the above diet plan and users need to provide start and end dates to activate this.

Nutritionist User: These are those users who will use Web UI App. They can create and modify food items, recipes, small CRUD forms to support LOV in application.

User Profile: These are those users who will use Mobile App. They login or sign up into the mobile/web application and provide necessary information to create a customized diet plan for their child user. This user does not contain any diet plan. This user must contain a child user and diet plans are applied on child users. For accurate recording of nutritional facts they need to confirm their meal intake from their daily diet plan in a simple and user friendly interface.

Child User: These are those users who are not directly using our application. They are operated by their guardians. “User Profile” can create multiple childs. Each child user has its own properties, e.g profile, weight, height, BMI, diet plan etc.

Sections of Web Admin application

Diseases: This is a simple CRUD form to manage LOV for diseases.

Procedures: This is a simple CRUD form to manage LOV for Procedures.

Milk Type: This is a simple CRUD form to manage LOV for milk types.

FoodAllergy: This is a simple CRUD form to manage LOV for Food Allergies.

Stomach Issues: This is a simple CRUD form to manage LOV for stomach issues.

Food Item Category: This is a simple CRUD form to manage LOV for food item category.

Recipes Category: This is a simple CRUD form to manage LOV for recipes category.

Serving Type: This is a simple CRUD form to manage LOV for serving types, e.g oz, cup, grams etc.

Serving SubType: This is a simple CRUD form to manage LOV for subtypes of serving types e.g cup has small,medium and large subtypes.

Nutritional Supplements: This is a simple CRUD form to manage LOV for nutritional supplements.

Food Items: This is a CRUD form with one detailed view about its attributes. Food Items are each individual item which we used to eat e.g Banana. Every food item has its description with Rich text, image, category, status, serving type, serving sub type, serving size and nutritional facts.

Serving size will be in grams or ml.

Recipes: This is a CRUD form with one detailed view about its attributes. Recipe is a group of food items which we used to eat e.g Banana Shake, Every recipe has its description with Rich text, image, category, status and primary nutritional facts.

Food Time: This is a simple CRUD form which is used to manage Food Times, It has a Food time name, default start time and end time.

Food time For User Child: This is a CRUD form which manages to add custom start and end time for each food item associated with the user.

Diet Plan: This is a CRUD form which manages to add a diet plan. Admin users will create multiple diet plans and after that he will link those plans with users. This feature is composed of two steps, in the first step the user will provide a No of days and list of Food times, then the system will create pre-defined empty entries for each day, In the second step, the user will select recipes for food times for each day. Please see document ***Web_Mocks_App_V1.0.0 (Diet Plan 6.5 section and its sub sections)***

Diet Plan User: This is a CRUD form which manages to add a diet plan for the user child. Admin users will create this form .This feature is composed of two steps. In the first step the user will select the user child, pre pre-created diet plan, Start and End Date. Then the system will create a day wise plan for selected user children according to their diet plan. In the second step, admin users can customize day wise. Please see document ***Web_Mocks_App_V1.0.0 (Diet Plan 6.6 section and its sub sections)***

Administrator User: This user is the custodian of a web ui app.

Sections of General Public Application

Overview:

This application is used by any person who wants to search recipes, diet plans, awareness in terms of blogs and recommended diet plans according to their health goals. They can also create a diet plan by themselves. This application also helps to manage multiple profiles, e.g. a mother wants to monitor and manage the diet of her two children along with herself. So she will create three profiles in the application. All business logic and data storage is handled in EatBaby API. Front-end is developed separately for Web and Mobile. There are some changes in the layout of mobile and web, but apis methods are the same for both.

Mobile application is a hybrid application, It stores some data in a local database and regularly pushes and pulls data from the server. This application is divided into multiple parts which are described below.

Note: To fully understand this section, please also take a look at design files in the “Design” folder of the drive Mobile_Sitemap_App_V1.0.0.pdf in the “Mobile” folder. Every heading below in the General Public application section represents a set of screens. You can identify them by their names and serial #.

E.g Below section 2 Profile is representing 4 screens in the design files. e.g 2.1, 2.2, 2.1.1

Food Times:

We have six types of food times in this application. These are breakfast, early snacks, lunch, evening snacks, dinner, and midnight snacks.

Each diet plan is based on these food times, there are some cases where a diet plan requires a person to take three meals a day then our food times for that diet plan would be Breakfast, Lunch and Dinner.

Developer Notes: We are providing authority to the users to change these food times names, e.g one user can change the name of Breakfast to Early Meal.

Menu:

Application is divided into below menu items. Option 1 and 2 contains some other navigation tabs:

1. Home / Dashboard (Profile, Calorie Target and Nutrition Goal)
2. Diet Plan (Recommended Diet Plans, Active Diet Plans, Created Diet Plans)
3. Blogs
4. Profile
5. Grocery
6. Recipes
7. Food Items
8. Messages
9. Subscription

1. Login and Sign up:

Login: In this form users can login into the application by the username and password, facebook and Gmail. And it must remember his details for the next login. E.g. the mobile user has logged in via facebook then for next time application will not ask for the login if facebook is already logged in on the mobile.

Sign Up: In this form users can signup with email or mobile along with password, or they can also use facebook and Gmail for signup. For facebook and Gmail, we will only ask for Email and the Profile name. On successful signup using facebook and Gmail we will save Email and the profile name in the user table. In case of signup from Email or mobile, we need to send OTP by email or phone respectively. Once a user confirms the OTP then he can access the application. Code will be generated for 60 minutes and users can resend the code after every 3 minutes.

2. Profile:

This form is used to save the profile information of the person whose diet is needed to be monitored and managed. This form is used at time of signup or the user can modify this form from the home. When this form is open from home then it will act as an edit profile while at time of sign up it will be a create form.

This form can also be accessed to create more than one profile.

Users will provide detailed information about their weight and height (weight should be taken in Kg and Pounds while height should be taken as inches and cm), other inputs are Disease, Surgery, Milk type, Food allergy, stomach issues, nutritional supplements (all these inputs can be multiple selected).

3. Growth Chart

In the profile section, users will provide information about weight and height. We will use those values and show their current bmi value. Possible display values are Overweight, Normal and Underweight.

Designer Notes: *We can show an image with these values.*

3.1 Calories Target

In this feature the patient will choose their target, they can choose to increase weight, decrease weight or maintain weight.

Designer Notes: *We will use radio buttons for this group.*

Users can select whether they need to increase their protein intake by using a checkbox.

Designer Notes: *We will use a checkbox for this input.*

If users select in (2.5) to increase weight then the **3.1.1** screen will open.

Users will decide whether they want to increase weight by below options

- 200-300 grams / week
- 500 grams / week (recomme\nded)
- 1 lbs / week
- 2Lbs / month
- Custom target.

Designer Notes: *We will use radio buttons to select one option. If they select a custom then we need to show them a textbox to enter a number of grams for a week.*

If users select in (2.5) to decrease weight then the **3.1.2** screen will open.

Users will decide whether they want to decrease weight by below options

- 200-300 grams / week
- 500 grams / week (recommended)
- 1 lbs / week
- 2Lbs / month
- Custom target.

Designer Notes: *We will use radio buttons to select one option. If they select a custom then we need to show them a textbox to enter a number of grams for a week.*

If users select in (2.5) to increase protein then the **3.1.3** screen will open.

Users will decide whether they want to decrease weight by below options

- 1 gram / body weight
- 1.2 gram / body weight
- 1.3-1.4 gram / body weight

- 1.5 gram / body weight
- Custom target.

Designer Notes: *We will use radio buttons to select one option. If they select a custom then we need to show them a textbox to enter a number of grams for a week.*

Developer Notes: *Screen 3.1.3 can be used after 3.1.1 or 3.1.2. If users want to increase their weight and also increase their protein intake then first we will show them screen 3.1.1 then 3.1.3.*

4. Nutritional Goal

After setting calories targets, then the application will calculate the calories, proteins, carbohydrates, fats, minerals and vitamins. This screen is only used to view data.

Designer Notes: *There are two sections of nutritional stats, one is composed of Calories, Proteins and Fats. Second section is composed of Different vitamins and minerals. We need to highlight the first part.*

5. Diet Plan Selection:

After calculating the goals, the user will decide whether he wants to use a saved diet plan (a menu for each age will be saved) or he wants to make a new plan. There is another option where users can ask a professional dietitian to help him to make the plan. This section is compose of below sub sections:

1. Created Diet Plans
2. Recommended Diet Plans
3. Active Diet Plans

5.1 Create New Plan

There are three steps to create a diet plan for a user's child. First you need to define a diet plan. Second is to choose recipes for everyday and third is to apply that plan over a period of time.

5.1.1 Update Diet Plan for Day

Topmost is the day count and day name mentioned with the previous and next option. There will be an option that a user can plan a menu for different days. Users will use these next and previous buttons to iterate between days. This page is divided into two parts, first is the data view part and second is the data input part.

Second part is about the selection of recipes against different food-times, users can select multiple recipes.

At the end of the page an option will be available whether the user wants to save this meal for one specific day (day will be mentioned in the drop down option, and the user can either choose one day or multiple days) or he can choose for every day.

Developer Notes: *In the first part we are showing comparison of nutritional values for nutritional goals and nutritional values of selected recipes. While in the second part we are taking recipes for different food items. Recipe selection is a multi select control with search option, you need to design all states of this control.*

5.1.1.1 Apply Plan

This form is used to create a diet plan for specific user children and dates.

Developer Notes: *When this form is called from 5.1.1 then we only need to display the selected user child in the user child dropdown and the diet plan which the user created in 5.1.1 in diet plan dropdown. There should be no other value displayed in these dropdowns except above ones.*

5.2 Load a Diet Plan

This screen will be used to display already created verified diet plans. This screen is composed of a list of diet plans with its title, food times, days and two lines of description. After every two lines of description there should be a show more button, when this button is pressed then it will show full description. This screen will show 5 diet plans when rendered for the first time. There should be a "show more" button at the bottom of the page which will load other diet plans.

There is a button in front of all diet plans titles, when the user clicks on this button then it will redirect to 5.1.1.1.

Diet Plan title is clickable, when the user clicks on title, it will redirect to 5.2.1.

5.2.1 Diet Plan Details

This screen shows all day wise details of previously selected diet plans with all the meals. The description includes the title and two lines of the description. There should be a "show more" button after a brief description then the menu of all the days is displayed with only basic food items (without ingredients and quantity).

5.2.1.1 Diet Plan Day Details

The screen shows the detail of diet plans with title, number of meals, number of times the menu will be used in a month , complete detail of the meal with food ingredients and quantity.

6. Active Diet Plan

The screen shows the diet plan title, number of meals on that day and number of days the diet plan will be used in a month. The description includes two lines by default. There

should be a “show more” button after a brief description , then the amount of nutrients required and consumed are mentioned (required/taken) with arrows displaying whether the user is consuming more or less than the required nutrients. Nutrients displayed includes calories, carbohydrates, proteins, fats, dietary fibre, saturated fatty acidsTotal monounsaturated fatty acids, Total polyunsaturated fatty acids, omega 3 fatty acids, trans fatty acids, Cholesterol, Calcium (Ca), Chromium (Cr), Chloride (Cl), Cobalt (Co), Copper (Cu), Fluoride (F), Iodine (I), Iron (Fe), Lead (Pb), Magnesium (Mg), Manganese (Mn) Mercury (Hg), Molybdenum (Mo), Nickel (Ni), Phosphorus (P), Potassium (K), Selenium (Se) Sodium (Na), Sulphur (S), Tin (Sn), Zinc (Zn), Vitamin A, Thiamin (B1), Riboflavin (B2), Niacin (B3), Pantothenic acid (B5), Pyridoxine (B6), Biotin (B7), Cobalamin (B12), Total folates and Vitamin C.

Fats, Vitamins and Minerals are clickable buttons, when user click on this text, it will redirect to 6.2

Designer Notes: *Insight section contains an arrow, you need to develop two color arrows, it might be green and red.If arrow is upward then it will be green and if arrow is downward then it will be red.*

6.1 Active Diet Plan Details

This screen shows all day wise details of the active diet plan with all the meals day by day. This page starts with the plan title, number of meals on that day and number of times the menu will be used in a month. This screen displays all the menus planned with the day, date and amount of nutrients required and consumed are mentioned (required/taken) with arrows displaying whether the user is consuming more or less than the required nutrients.

6.2 Details of Vitamins/ Fats and Minerals

This screen shows the comparison of required and taken nutrients.

Designer Notes: *Every row must have an arrow with green or red color.*

7. Created Diet Plan

The screen shows the diet plans created by the user. The information mentioned on the screen includes diet plan title, number of meals in a day, number of times diet plan will be used in a month and two lines of description of each plan created.

Designer Notes: *This screen is a copy of section 5. It only contains an update button.*

7.2 Diet Plan Details

This screen shows all the created diet plans with all the meals . The description includes the title and two lines description. There should be a “show more” button after a brief description then the menu of all the days is displayed with only basic food items (without

ingredients and quantity). An “update” button with the title will help to update any meal, food items and quantity in the respective diet plan.

Developer Notes: *When the user clicks on the update button then it will redirect to screen 5.1.1.*

7.2.1 Diet Plan Day Details

The screen shows the detail of created diet plans with title, number of meals, number of times the menu will be used in a month , complete detail of the meal with food ingredients and quantity. An “update” button with the title will help to update any meal, food items and quantity in the respective diet plan.

Developer Notes: *When the user clicks on the update button then it will redirect to screen 5.1.1.*

8. Dashboard

Dashboard includes the insight of that specific date by showing the amount of nutrients required and consumed are mentioned (required/taken) with the percentages. Nutrients displayed include calories, carbohydrates, proteins, fats, Vitamins and minerals.

The screen will further display the diet plan of the day with complete detail of the meal with food ingredients and quantity. This screen also has a next and previous button which will load details of that specific day.

8.1 Update meal status of the day

This screen will be used when the user wants to add the meal intake in terms of percentage for a complete day. If the user chooses 50% then it means he/she has intake of 50%. This screen will have 4 options for every meal of the active diet plan. The user can choose whether he consumed 0% / 25% / 50% /75% /100% of the specific meal. This will help to give the insight of the nutrients consumed throughout the day.

8.2 Update meal status of specific meal

The screen will have 4 options for the selected meal of the active diet plan. The user can choose whether he consumed 0% / 25% / 50% /75% /100% of the specific meal. This will help to give the insight of the nutrients consumed throughout the day for a single meal.

9. Change Profile

The screen displays the different profiles through which the user has logged in the application. The screen shows the name, age and an option whether the profile diet plan is active or inactivated. The button “select” will help the user to select the profile.

At the bottom there is a button “add a new child” to add another profile.

10. Grocery list

The screen shows the name of the child and the dates till which the user wants to estimate the grocery items. The button " search " will show the name and quantity of the food items to be required in those dates that are chosen by the user.

11. Recipes

Recipes of the food items available in the menu can be seen after clicking the specific food. The screen shows the name of the food item, picture, quantity and the amount of nutrients present in the food item.

12. Food items

The screen will display the food items, the quantity and the amount of nutrients present in the food item. Nutrients displayed includes calories, carbohydrates, proteins, fats, dietary fibre, saturated fatty acids, Total monounsaturated fatty acids, Total polyunsaturated fatty acids, omega 3 fatty acids, trans fatty acids, Cholesterol, Calcium (Ca), Chromium (Cr), Chloride (Cl), Cobalt (Co), Copper (Cu), Fluoride (F), Iodine (I), Iron (Fe), Lead (Pb), Magnesium (Mg), Manganese (Mn), Mercury (Hg), Molybdenum (Mo), Nickel (Ni), Phosphorus (P), Potassium (K), Selenium (Se), Sodium (Na), Sulphur (S), Tin (Sn), Zinc (Zn), Vitamin A, Thiamin (B1), Riboflavin (B2), Niacin (B3), Pantothenic acid (B5), Pyridoxine (B6), Biotin (B7), Cobalamin (B12), Total folates and Vitamin C. By clicking the right button the user can add the food item in the favorite tab.

13. Payments Mechanism:

1. Free Package
2. Basic Package
3. Standard Package

13.1 Free Package: In this package basic application is available for 30 days, These number of days will be populated by Database.It'll include following major features:

a. One child allowed
b. Adds included

13.2 Basic Package: In this package all features of the application are available to users and they can create their recipes and diet plan by themselves.It'll include following major features:

a. Multiple children allowed
b. Adds free
c. View grocery list

13.3 Standard Package: This package contains all features of Basic Package and 2 monthly calls with professional dietitians and diet plans will be created by Dietitians. It'll include following major features:

a. Multiple children allowed
b. Adds free
c. View grocery list
d. 2 calls per month with diettitions
e. Monthly follow-ups

14. Coupons

Coupons can be created for Basic and Standard Package along with validity time and time duration. E.g we have created a Coupon for Basic package, we need to add the date by which that coupon can be activated and after that date the coupon code will be deactivated. and the duration in days by which Package will be active.

Coupon code will be 6 characters. 2 numbers and 8 characters. E.g A5BCDEFGH8

14.1 Flow of Events

Whenever a user signup takes place, then after completing the user child profile user will be redirected to the calorie target view.

After setting the calorie target, the user will be redirected to a view called Subscription View. Here, the user will view a list of options for package subscription along with packages' features. An input field for coupon code will become active as soon as the user selects the option of using coupon.

If a user selects a package, then the system must redirect the user to the payment gateway. If a user selects the use coupon option and enters a coupon code, then the system must verify whether it's a valid coupon code or not by checking the code in the database and also by checking the current date whether it lies in the date range of the coupon or not. If it's verified, then the system must redirect the user to the payment gateway. After successful payment transaction user subscription will be successful, and the user can access the features of the subscribed package. If not, the system must ask the user to enter a valid coupon code.

If the user doesn't select a package, then the free package will be subscribed for the user and the user will then get access to the features of the free package.

On successful login, the system checks the users' subscribed package and gives access to just those features of the app that the package allows.

If users' package (basic or standard) is expired then if the user has more than one child then the user can not view the profiles section where he/she can add more than one child.

On login, the system must check the subscription details of the user. If it's using some coupon, the date range of the coupon is checked whether the current date lies in that range or not. If not, the free package will be automatically subscribed for the user. If yes, the user will be redirected to the dashboard. If it's not using a coupon then it's package date range will be compared to the current date. If it's a valid date, then users get access to the package features. If not, the free package will be automatically subscribed for the user.

Whenever a user logins it must be checked at the backend whether the user is using some coupon or not. If yes, then the current date must be compared with the valid date range for the user's coupon. If the coupon is valid then the user must see the relevant controlled access view depending upon the coupon and then the package type. If it's invalid, then the user must be redirected to the subscription menu for a new subscription or use a new coupon. If not, then the user subscription package's date range must be checked. If it's valid, then the user will be redirected to the dashboard.If it's invalid, then the free package will be automatically subscribed for the user.

Public Website:

15. Questions

The system must provide a module that will allow users to ask questions in public mode.

The system must allow users to ask and search questions about various categories. It must allow users to comment, rate ,like, and dislike questions and comments. It must validate the form of a question and the comment before posting it. The system must keep a record of the questions asked by a particular user and allow users to view their previously asked questions. While asking a question, user has the ability to hide their identity, which means user can post their question anonymously.

Questions Module Landing/ List Page:

Question Module page was split into two sections such as the following

- **Browse Section:**

This section lists all categories available in the system with the number of questions asked under that category. This section will also shows the following additional navigation

➔ **My questions:** This navigation is available which means dynamically visible if the user asked any question before and this navigation lists out those questions for the user to view on it.

- **Questions Section:** This section shows the list of questions available under the speciality category selected from the 'Browse Section'

MY QUESTIONS

- This section dynamically visible if the user asked any question before
- This section shows all the questions (both normal question and second opinion question) the user has asked
- For free question asked, user can have the ability to do the following

View answer

Rate answer

Comment on answer

Question Details Page:

The main aim of this page is to allow users to view the complete details of the question along with answers received for that question. There are the following two ways user will be redirected to this page

1. When clicking any specific question from "Questions List Page"
2. When clicking 'View Answer' option from the email notification they received for the question they asked

In this page, user can see the complete details about that question including the following

- Question Header
- Question Details
- Asking User information
- Answers List : For each answer, user can see the following information
  - ❖ Answer text
  - ❖ Answering Provider information
  - ❖ Answering Provider Rating

The following navigation option also displayed in Question details page based on the user type

- Ask a Question
- Answer a Question
- Rate Answer
